# Association between diet quality and malnutrition: pooled results from two population-based studies in older adults

**DOI:** 10.1186/s12877-024-04984-5

**Published:** 2024-05-10

**Authors:** Alba Marcos-Delgado, Humberto Yévenes-Briones, Tania Fernández-Villa, Vicente Martín-Sánchez, Pilar Guallar-Castillón, Fernando Rodríguez-Artalejo, Esther Lopez-Garcia

**Affiliations:** 1 Department of Biomedical Sciences, Area of Preventive Medicine and Public Health, Universidad de León, León, Spain; 2 The Research Group in Gene-Environment and Health Interactions, Institute of Biomedicine (IBIOMED), Universidad de León, León, Spain; 3 School of Medicine, Department of Preventive Medicine and Public Health, Universidad Autónoma de Madrid, Avda. Arzobispo Morcillo 2, Madrid, 28029 Spain; 4 CIBER in Epidemiology and Public Health (CIBERESP), Madrid, Spain; 5 IMDEA-Food Institute, CEI UAM+CSIC, Madrid, Spain

**Keywords:** Diet quality, Mediterranean diet, Alternative healthy eating index, Malnutrition, GLIM criteria, Older adults

## Abstract

**Background:**

The role of diet quality on malnutrition in older adults is uncertain, due the paucity of the research conducted and the use of use of screening tools that did not consider phenotypic criteria of malnutrition.

**Objective:**

To evaluate the association of two indices of diet quality, namely the Mediterranean Diet Adherence Screener (MEDAS) and the Alternative Healthy Eating Index (AHEI-2010), with malnutrition among community-dwelling older adults in Spain.

**Methods:**

Cross-sectional analysis of data from 1921 adults aged ≥ 60 years from the Seniors-ENRICA-1 (SE-1) study, and 2652 adults aged ≥ 65 years from the Seniors-ENRICA-2 (SE-2) study. Habitual food consumption was assessed through a validated diet history. Malnutrition was defined according to the Global Leadership Initiative on Malnutrition (GLIM) phenotypic criteria. Statistical analyses were performed with logistic regression with adjustment for socioeconomic and lifestyle variables as well as for total energy and protein intake.

**Results:**

The prevalence of malnutrition in the SE-1 study was 9.5% (95% confidence interval: 8.2 to 10.9) and 11.7% (10.5 to 13.9) in the SE-2. Adherence to the MEDAS score was associated with lower prevalence of malnutrition [pooled odds ratio for high (≥ 9 points) vs. low adherence (< 7 points): 0.64 (0.48–0.84); p-trend < 0.001]. Higher adherence to the AHEI-2010 also showed an inverse association with malnutrition (pooled odds ratio for quartile 4 vs. 1: 0.65 (0.49–0.86); p-trend 0.006). Among the individual components, higher consumption of fish and long-chain n-3 fatty acids in MEDAS and AHEI-2010, and of vegetables and nuts and legumes in AHEI-2010, and lower intake of trans-fat and sugar-sweetened beverages and fruit juice in AHEI-2010 were independently associated with lower odds of malnutrition.

**Conclusion:**

Adherence to high diet-quality patterns was associated with lower frequency of malnutrition among older adults.

**Clinical trial registry:**

ClinicalTrials.gov identifier: NCT02804672. June 17, 2016.; ClinicalTrials.gov NCT03541135. May 30, 2018.

**Supplementary Information:**

The online version contains supplementary material available at 10.1186/s12877-024-04984-5.

## Introduction

Malnutrition is a syndrome characterized by energy-protein undernutrition [1]. In older adults, malnutrition has been associated with increased morbidity and mortality [2], sarcopenia [3], frailty [4], lower health-related quality of life [5], and important healthcare costs [6]. In addition, underdiagnosis and under-treatment of malnutrition are common in an increasingly aging population [7]. Unfortunately, there is not much data on its prevalence in community-living older adults [6, 8, 9]. This may be due to varying definitions of malnutrition and different diagnostic criteria [3]. Recently, a consensus has been reached to establish a common definition, known as the Global Leadership Initiative on Malnutrition (GLIM) criteria [1], which facilitates comparing results across different studies.

Malnutrition has multiple causes, including older age, living alone, impaired physical function, and a previous hospitalization [10]. Other contributing causes are aging-associated disorders, such as loss of smell, taste and appetite, mastication problems, dysphagia, or the alteration of the physiological mechanisms of thirst, as well as primary diseases that affect nutritional status [11]. Dietary strategies to prevent malnutrition have largely focused on nutrient supplementation [12]. However, there is increasing evidence suggesting that an adequate nutrient intake obtained from the habitual diet could prevent the development of physical impairment and frailty, and possibly, malnutrition [13, 14]. Since dietary recommendations based on diet patterns are easier to implement in the population [15], the identification of those with most benefit for the older population is of great interest to prevent malnutrition.

Two well-known healthy dietary patterns are the Mediterranean diet, assessed by the MEDAS score, and the Western healthy diet, represented by Alternative Healthy Eating Index (AHEI) 2010. Higher adherence to the MEDAS and AHEI-2010 scores have been associated with lower risk of chronic diseases, including cardiovascular disease [16, 17], type 2 diabetes [18] or cancer [19, 20], and death from non-traumatic diseases [21]. In addition, there is evidence that these diet patterns can prevent the frailty syndrome in older adults [22]. However, a recent cross-sectional study among Chinese population found that higher adherence to the Dietary Quality Index International, but not to the Mediterranean diet, was associated with lower likelihood of malnutrition based on the GLIM criteria [23]. Other studies have assessed different diet quality scores in relation to other definitions of malnutrition, with inconsistent results [24–27].

Therefore, the objective of our study was to evaluate the association between adherence to the MEDAS score and AHEI-2010 with malnutrition, as measured by the GLIM criteria, in two studies of community-dwelling older adults in Spain.

## Methods

### Study design and participants

This study used data from the Seniors-ENRICA-1 (SE-1) and Seniors-ENRICA-2 (SE-2) studies. The SE-1 was established in 2008–2010 as a cross-sectional study of a representative sample of the population of Spain aged ≥ 18 years [28]. Of them, 3289 participants aged ≥ 60y comprised the Seniors-ENRICA cohort. Information on sociodemographic data, lifestyle, health status, and morbidity were collected by a computer-assisted telephone interview. In two subsequent home visits, a physical examination was done to obtain anthropometric data. In 2012, data were updated and a battery of tests of physical and cognitive function was included; data from this wave have been used in these analyses since the physical assessment included is more comprehensive than in the baseline wave. Participants gave informed written consent, and the Clinical Research Ethics Committee of the *La Paz* University Hospital in Madrid approved the study (PI-2144, PI-3554).

The SE-2 study included individuals who were recruited in the years 2015–2017 through a non-probabilistic sample of convenience, with stratified cluster sampling by sex and district among all individuals aged 65 years or older with a national healthcare card, from community-dwelling residents in the city of Madrid and four surrounding towns. Baseline data were collected with a similar protocol to that used in the SE-1 [29, 30]. Study participants gave written informed consent, and the study was approved by the Clinical Research Ethics Committee of the *La Paz* University Hospital in Madrid (PI-1793).

### Study variables

#### Diet

Food consumption was obtained by trained interviewers through a validated computerized diet history, which was developed from the one used in the EPIC-Spain cohort study [31, 32]. Habitual consumption of 860 foods and beverages consumed up to 1 time every 15 days was recorded, to complete a standard 1-week of consumption. Portion sizes, cooking methods, degree of food processing, and weekly and seasonal variations in food consumption were also recorded. Nutrient intakes were derived from Spanish food composition tables [32]. The validity of the diet history was evaluated by comparing its results with seven 24-h recalls over a one-year period among a subsample of participants; the observed correlations ranged between 0.27 and 0.71 across food groups and nutrients [32], which are in line with those for most instruments assessing self-reported diet in population studies [33].

Two healthy diet patterns were derived: the Mediterranean Diet Adherence Screener (MEDAS) and the Alternative Healthy Eating Index (AHEI-2010). The MEDAS score measures adherence to the Mediterranean diet in the Spanish population [34], and comprises 14 items, where 12 of them refer to the frequency of consumption of several foods, and another 2 to dietary habits typical of the Mediterranean diet in Spain. The items are scored 0 or 1 according with the compliance with the cut-off point; the global score ranges from 0 (lowest) to 14 (highest adherence) (Table S1).

The AHEI-2010 was based on a comprehensive review of foods and nutrients that had consistently been associated with lower risk of chronic disease in clinical and epidemiological investigations [35]. A value of 10 is assigned to higher consumption of healthy foods and nutrients, while a value of 0 is assigned to higher consumption of unhealthful dietary components. Intakes between the minimum and maximum levels are scored proportionately. The global score ranges from 0 (lowest) to 110 (highest diet quality) (Table S1).

#### Malnutrition assessment

We defined malnutrition according to the criteria by the Global Leadership Initiative on Malnutrition (GLIM) to provide a measure of this condition encompassing risk screening and diagnosis [1]. The GLIM group established two groups of criteria: phenotypic, including non-volitional weight loss, low body mass index (BMI), and reduced muscle mass; and etiological, indicating that malnutrition is due to chronic disease with inflammation, acute disease with severe inflammation, or gastrointestinal condition that adversely impacts food assimilation or absorption.

Non-volitional weight loss was defined as positive response to the question: “Have you lost 4.5 kg of weight or more in the last year?” [36]. Weight and height, measured under standardized conditions, using electronic scales (model Seca 841, precision to 0.1 kg) and portable extendable stadiometers (model Ka We 44 444Seca). Mean values of two measurements were used for the analyses. BMI was calculated as weight in kg divided by squared height in m. Participants were classified as “with low BMI” if < 20 kg/m^2^ among those aged < 70 years, or if < 22 kg/m^2^ among participants ≥ 70 years [1].

Muscle mass was measured with bioelectrical impedance analysis (BIA) (Tanita® SC-240MA, Tanita Corp., Tokyo, Japan). Skeletal muscle mass (SMM) (kg) was calculated with the equation developed by Janssen et al. [37]: ([height^2^/resistance * 0.401] + [sex * 3.825] + [age * -0.071]) + 5.102, where height is given in cm, resistance in ohms (from BIA), sex as 1 for males and 0 for females, and age in years. Skeletal muscle mass index (SMI) was estimated by dividing SMM by height in meters squared. Sex-specific thresholds for reduced muscle mass were < 7.26 kg/m^2^ for males, and < 5.25 kg/m^2^ for females [38]. We defined moderate-to-severe malnutrition as having at least one phenotypic criterion, since only the phenotypic criteria are proposed for severity grading, and since both studies included community-dwelling population, without acute etiological criteria that needed constant supervised care.

#### Other variables

In 2012 for the SE-1 and 2015–2017 for the SE-2, participants reported their sex, age, educational level, smoking status, alcohol consumption, sedentary behavior, and total energy intake. Physical activity was assessed with the validated questionnaire developed in the EPIC cohort study in Spain; participants were asked for the number of hours that they spent in a typical week during the last year on each of the following activities: walking, cycling, gardening, do-it-yourself activities at home, playing sports (running, fitness, aerobics, swimming, soccer, tennis, etc.), and climbing stairs. Total physical activity, in metabolic equivalent tasks (METs h/week), was derived from this information [39]. Sedentariness was approached by the time watching TV (h/week). Lastly, the following physician-diagnosed diseases were self-reported: musculoskeletal disease, cardiovascular disease, diabetes, cancer, chronic lung disease, and depression requiring treatment.

### Statistical analyses

Of the 3289 participants from SE-1 in 2012, a total of 1921 provided valid data on diet and the variables to create the GLIM criteria. In SE-2, 2652 out from 3273 participants provided information on diet and malnutrition and were included in the analyses. Participants excluded in both cohorts were older, more often women, with lower educational level, with more comorbidity, and reporting less physical activity and more sedentary time per week than participants who accepted to be examined.

The prevalence and 95% confidence interval (CI) of malnutrition and each of its component elements were calculated in both studies. Differences in sociodemographic lifestyle and clinical characteristics by malnutrition status were assessed using the chi-square test for categorical variables and analysis of variance (ANOVA) test for quantitative variables.

Participants were classified into three groups according to their adherence to the diet quality indexes: for the MEDAS, as low (< 7 points), moderate (≥ 7 to < 9 points), and high (≥9 points); for the AHEI-2010, in quartiles. We used logistic regression models to calculate odds ratios (OR), and their 95% CI for the association between the diet indexes and malnutrition. Four consecutive multivariable models were built. The first one was adjusted for sex and age; the second model was additionally adjusted for educational level (primary, secondary and university), as a *proxi* of socioeconomic level, leisure-time physical activity (METs-h/week), time spent watching TV (h/week), as a *proxi* of sedentariness, and smoking status (current-, former- or never- smoker). The third model also included energy intake (kcal/day) and total protein intake (g/day). A fourth model was also adjusted for morbidity (musculoskeletal disease, cardiovascular disease, cancer, chronic lung disease and depression). The lowest category of adherence to the MEDAS and the first quartile in the AHEI-2010 were considered as the reference in all the models. We also examined each diet quality score as a continuous variable (per 1-SD) in association with malnutrition. We performed these analyses in each study and then pooled the results to obtain a summary OR estimate, by using inverse variance-weights and a random-effects model [40], which allowed for between-study heterogeneity.

We performed stratified analyses by sex, age, educational level, smoking status, energy intake, total protein intake, as well as presence of musculoskeletal disease, cardiovascular disease, and depression, to better understand their contribution to the examined association. Of note, physical activity, and hours of watching TV were not considered in the stratified analyses since we observed in the multivariable models that their impact modifying the study association was not as relevant as the other variables included. To test for interactions, we used likelihood-ratio tests to compare models with and without an interaction term, defined as the cross-product of the diet quality index (as continuous variable) and the stratification variable. In addition, we examined the association of each component of the diet quality indexes with malnutrition by using fully-adjusted multivariable models that were also adjusted for each of the other food components of the indexes. Finally, a sensitivity analysis was performed excluding the alcohol component from the MEDAS and AHEI-2010 to better understand its role on the studied association. Statistical significance was set at two-tailed *p* < 0.05. Analyses were performed with Stata (version 16.1; Stata Corp., College Station).

## Results

The prevalence of malnutrition was 9.5% (95% CI: 8.2 to 10.9) in the SE-1 study and 11.7% (10.5 to 13.9) in the SE-2 (Table 1), with non-volitional weight loss being the most prevalent criterion in both studies.

**Table 1:** Malnutrition prevalence according to the GLIM criteria in the Seniors-ENRICA-1 and Seniors-ENRICA-2 studies

	Prevalence, %	95% confidence interval	
Seniors-ENRICA-1 (*n* = 1921)			
Malnutrition ^a^	9.5	8.2, 10.9	
Low body mass index ^b^	1.5	1.0, 2.1	
Weight loss ^c^	7.6	6.5, 8.9	
Reduced muscle mass ^d^	0.9	0.6, 1.5	
Seniors-ENRICA-2 (*n* = 2652)			
Malnutrition ^a^	11.7	10.5, 13.9	
Low body mass index ^b^	4.9	4.1, 5.8	
Weight loss ^c^	5.7	4.9, 6.7	
Reduced muscle mass ^d^	1.8	1.3, 2.4	

^a^ Requires at least 1 phenotypic criterion for diagnosis of malnutrition

^b^ <20 kg/m^2^ if < 70 years, or < 22 kg/m^2^ if ≥ 70 years

^c^ Non-volitional weight loss ≥ 4.5 kg in the last year

^d^ Appendicular skeletal muscle index < 7.26 kg/m^2^ for men and < 5.25 kg/m^2^ for women

Main characteristics of the study participants are presented in Table 2. Compared to those without malnutrition, those suffering this syndrome were often women, of older age and lower educational level. In the SE-2 the prevalence of smoking was higher, and in SE-1 physical activity was lower among participants with malnutrition. Also, those with malnutrition showed a higher prevalence of musculoskeletal, cardiovascular disease and depression in the SE-1, and of cardiovascular disease in the SE-2. Lastly, those with malnutrition reported lower energy and protein intake.

**Table 2:** Participants’ characteristics according to malnutrition status in the Seniors-ENRICA-1 and Seniors-ENRICA-2 studies

	Seniors-ENRICA-1 (*n* = 1921)			Seniors-ENRICA-2 (*n* = 2652)		
	Malnutrition according to the GLIM criteria					
	No	Yes	*p* value ^a^	No	Yes	*p* value ^a^
Participants, n (%)	1739 (90.5)	182 (9.5)		2343 (88.4)	309 (11.7)	
Sex, men %	48.0	37.4	0.006	47.7	44.7	0.31
Age, y	71.5 (6.2)	73.7 (7.3)	< 0.001	71.5 (4.4)	72.5 (4.1)	< 0.001
Educational level, %						
≤Primary	52.2	64.8	0.005	63.7	66.3	0.56
Secondary	25.1	19.2		18.4	18.1	
University	22.7	15.9		17.9	15.5	
Smoking status, %						
Current	11.6	9.3	0.27	9.0	13.0	0.05
Former	30.2	26.4		39.1	34.3	
Never	58.2	64.3		52.0	52.8	
Leisure-time physical activity, METs-h/week	57.5 (31.5)	51.6 (29.7)	0.02	67.2 (36.1)	65.6 (36.9)	0.49
Time spent watching TV, h/week	19.5 (10.6)	19.5 (11.5)	0.97	22.7 (11.5)	22.0 (11.0)	0.31
Diagnosed morbidity, %						
Musculoskeletal disease^b^	48.3	56.6	0.03	44.4	48.2	0.20
Cardiovascular diseases^c^	6.2	13.2	< 0.001	6.3	9.7	< 0.001
Cancer	3.1	3.9	0.59	2.9	3.2	0.71
Chronic lung disease	9.6	8.2	0.57	7.9	7.8	0.94
Depression requiring treatment	8.1	15.9	< 0.001	8.4	8.7	0.82
Energy intake, kcal/day	2021 (447)	1925 (459)	0.006	1960 (350)	1903 (382)	0.009
Total protein intake, g/day	92.1 (22.8)	86.6 (21.3)	0.002	89.8 (16.8)	85.6 (17.8)	< 0.001
Total protein intake, g/kg/day	1.26 (0.3)	1.32 (0.3)	0.01	1.2 (0.3)	1.4 (0.3)	< 0.001
MEDAS score	7.6 (1.6)	7.1 (1.6)	< 0.001	7.2 (1.7)	7.0 (1.7)	0.14
AHEI-2010 score	62.9 (9.7)	59.9 (11.0)	< 0.001	62.7 (9.4)	62.1 (9.7)	0.27

Abbreviations: MET: metabolic equivalent; MEDAS: Mediterranean diet adherence screener; AHEI − 2010: Alternate Healthy Eating Index-2010

For continuous variables, mean and standard deviation are reported. ^a^*P*- value: Chi-square test was used for categorical variables and ANOVA test for quantitative variables. ^b^ Osteoarthritis, arthritis and hip fracture. ^c^ Ischemic heart disease, stroke, and heart failure

A higher MEDAS score was associated with lower prevalence of malnutrition in both studies (Table 3). After adjustment for age and sex, the pooled OR (95% CI) for high vs. low MEDAS adherence was 0.67 (0.51–0.88), *p*-trend 0.001. Additional adjustment for educational level, lifestyle, energy and protein intake and morbidity strengthened the association [model 4: OR 0.64 (0.48–0.84), *p*-trend < 0.001]. A higher adherence to the AHEI-2010 was also associated with lower odds of malnutrition; in model 4, the pooled OR (95% CI) for Q4 vs. Q1 was 0.65 (0.49–0.86), *p*-trend 0.006 (Table 4).

**Fig. 1:**
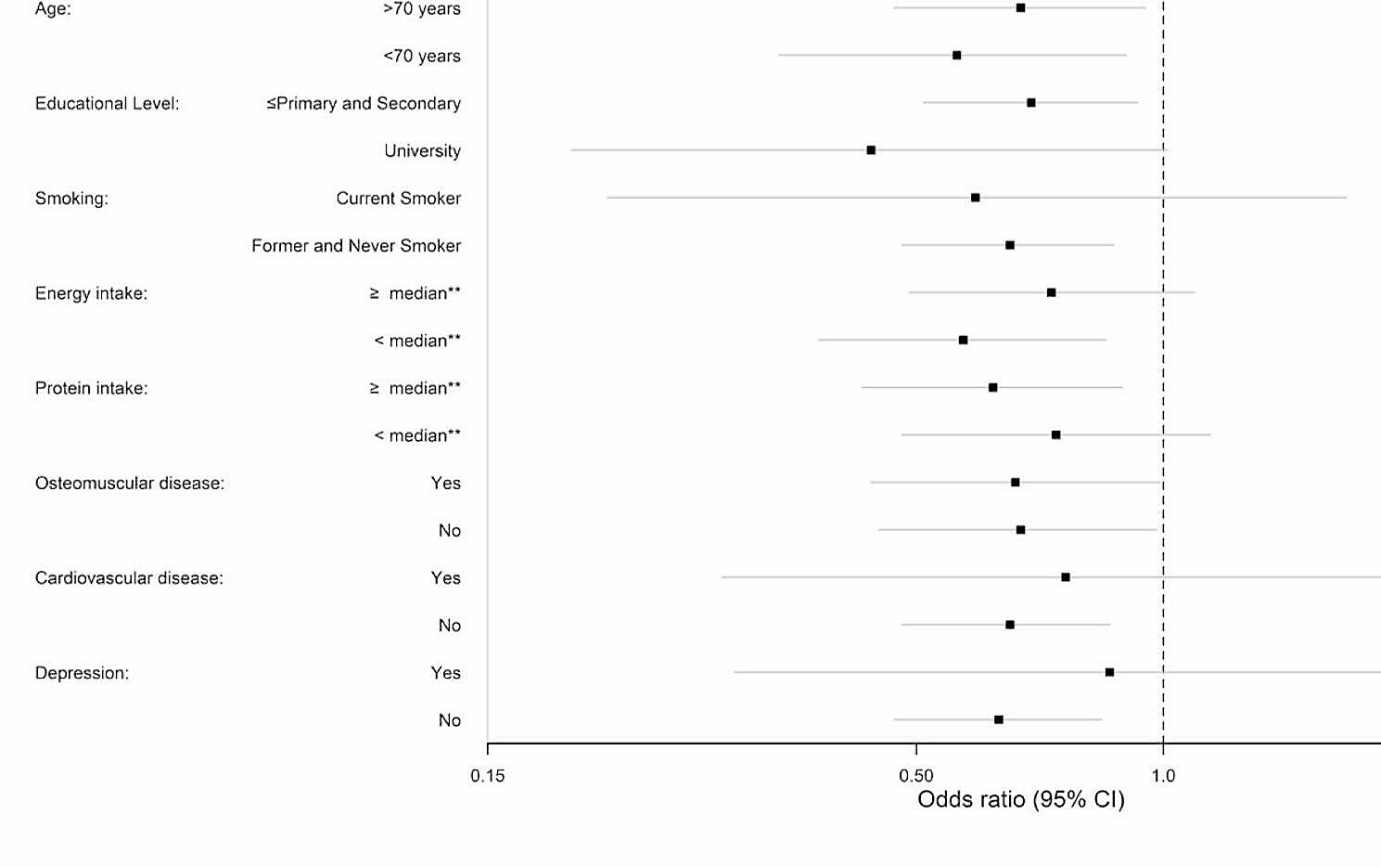
Pooled odds ratios (95% confidence interval) for the association between the MEDAS score (high adherence vs. low adherence) and malnutrition, stratified by characteristics of study participants. Logistic regression models adjusted for age, sex, educational level (≤primary and secondary or university), smoking status (current, former and never smoker), leisure-time physical activity (METs-h/week), time spent watching TV (h/week), energy intake (kcal/day), total protein intake (kg/d), and morbidity (musculoskeletal disease, cardiovascular diseases, cancer, chronic lung disease, and depression), except for the stratification variable. *P* for interaction in all comparisons were non-significant **Median of energy intake: 1948 kcal. Median of protein intake: 1.2 g/kg

**Fig. 2:**
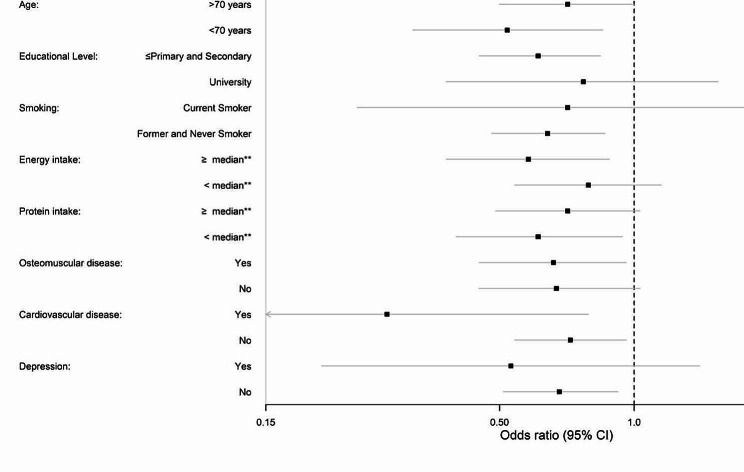
Pooled odds ratios (95% confidence interval) for the association between AHEI-2010 score (quartile 4 vs. quartile 1) and malnutrition, stratified by characteristics of study participants. Logistic regression models adjusted for age, sex, educational level (≤primary and secondary or university), smoking status (current, former and never smoker), leisure-time physical activity (METs-h/week), time spent watching TV (h/week), energy intake (kcal/day), total protein intake(kg/day), and morbidity (musculoskeletal disease, cardiovascular disease, cancer, chronic lung disease, and depression), except for the stratification variable. *P* for interaction in all comparisons were non-significant **Median of energy intake = 1948 kcal. Median of protein intake = 1.2 g/kg

In stratified analyses by characteristics of study participants, high versus low adherence to the Mediterranean diet was associated with malnutrition in all the strata except those with small sample size (e.g., current smokers and those with cardiovascular diseases or depression). The associations were slightly stronger among women, those < 70 years, with high educational level, and those who consumed less calories (*p* for interaction not significant in any case) (Fig. 1). For the AHEI-2010 the associations were somewhat stronger among men, those < 70 years, with lower educational level and higher energy intake (*p* for interaction not significant in any case) (Fig. 2).

Some individual components of the diet quality scores were associated with malnutrition. Higher consumption of fish and long-chain n-3 fatty acids in MEDAS and AHEI-2010, vegetables and nuts and legumes in AHEI-2010, and lower intake of trans-fat and sugar-sweetened beverages and fruit juice in AHEI-2010 were independently associated with lower odds of malnutrition in pooled data (Tables S2-S3). Lastly, sensitivity analyses excluding alcohol from the MEDAS score and AHEI-2010 indexes still showed an association between adherence to these diet patterns and lower odds of malnutrition (Tables S4-S5).

**Table 3:** Odds ratios (95% confidence interval) for the association between the Mediterranean Diet Adherence Screener (MEDAS) score and malnutrition according to the GLIM criteria in the Seniors-ENRICA-1 and Seniors-ENRICA-2 studies

	Adherence to the MEDAS score					
	Low	Moderate	High	*p* trend	Per 1-SD increment in MEDAS	
	< 7 points	7–9 points	≥ 9 points			
Seniors-ENRICA-1 (*n* = 1921)						
N	533	852	536			
n cases	64	85	33			
Model 1	1.00	0.84 (0.59, 1.19)	0.53 (0.34, 0.82)	0.003	0.78 (0.67, 0.92)	
Model 2	1.00	0.85 (0.60, 1.20)	0.55 (0.35, 0.86)	0.005	0.79 (0.68, 0.93)	
Model 3	1.00	0.81 (0.57, 1.16)	0.49 (0.31, 0.78)	0.001	0.77 (0.65, 0.90)	
Model 4	1.00	0.82 (0.57, 1.17)	0.49 (0.31, 0.77)	0.001	0.77 (0.65, 0.90)	
Seniors-ENRICA-2 (*n* = 2652)						
N	919	1166	567			
n cases	113	140	56			
Model 1	1.00	0.98 (0.75, 1.28)	0.78 (0.55, 1.10)	0.15	0.92 (0.81, 1.03)	
Model 2	1.00	0.98 (0.75, 1.28)	0.78 (0.55, 1.09)	0.14	0.91 (0.81, 1.03)	
Model 3	1.00	0.94 (0.71, 1.23)	0.75 (0.53, 1.06)	0.10	0.90 (0.80, 1.02)	
Model 4	1.00	0.93 (0.71, 1.22)	0.74 (0.52, 1.05)	0.08	0.89 (0.79, 1.01)	
Pooled*						
Model 1	1.00	0.93 (0.75, 1.15)	0.67 (0.51, 0.88)	0.001	0.87 (0.79, 0.95)	
Model 2	1.00	0.93 (0.75, 1.15)	0.68 (0.52, 0.89)	0.001	0.86 (0.79, 0.95)	
Model 3	1.00	0.89 (0.72, 1.11)	0.64 (0.49, 0.85)	< 0.001	0.85 (0.77, 0.94)	
Model 4	1.00	0.88 (0.71, 1.10)	0.64 (0.48, 0.84)	< 0.001	0.84 (0.77, 0.93)	

Model 1: logistic regression model adjusted for age and sex

Model 2: as in Model 1 and additionally adjusted for educational level (primary, secondary and university), smoking status (current, former and never smoker),

leisure-time physical activity (METs-h/wk), and time spent watching TV (h/wk)

Model 3: as in Model 2 and additionally adjusted for energy intake (kcal/day), and total protein intake (kg/day)

Model 4: as in Model 3 and additionally adjusted for morbidity (musculoskeletal disease, cardiovascular diseases, cancer, chronic lung disease, and depression)

*Pooled data: models combined using a random-effects model

**Table 4:** Odds ratios (95% confidence interval) for the association between the Alternate Healthy Eating Index-2010 (AHEI-2010) and malnutrition according to the GLIM criteria in the Seniors-ENRICA-1 and Seniors-ENRICA-2 studies

	Adherence to the AHEI-2010							
	Quartile 1	Quartile 2	Quartile 3	Quartile 4	*p* trend	Per 1-SD increment in AHEI-2010		
Seniors-ENRICA-1 (*n* = 1921)								
Range	< 56.1	56.1–62.9	63.0-69.3	≥ 69.4				
N	481	480	480	480				
n cases	68	37	43	34				
Model 1	1.00	0.49 (0.32, 0.74)	0.58 (0.38, 0.87)	0.47 (0.30, 0.72)	< 0.001	0.74 (0.64, 0.87)		
Model 2	1.00	0.48 (0.31, 0.74)	0.58 (0.38, 0.88)	0.47 (0.30, 0.73)	< 0.001	0.75 (0.64, 0.87)		
Model 3	1.00	0.47 (0.31, 0.73)	0.55 (0.37, 0.84)	0.47 (0.30, 0.73)	< 0.001	0.74 (0.63, 0.87)		
Model 4	1.00	0.48 (0.31, 0.74)	0.55 (0.36, 0.84)	0.48 (0.31, 0.76)	< 0.001	0.74 (0.63, 0.87)		
Seniors-ENRICA-2 (*n* = 2652)								
Range	< 56.2	56.2–62.9	63.0-69.3	≥ 69.4				
N	663	663	663	663				
n cases	81	72	88	68				
Model 1	1.00	0.86 (0.61, 1.21)	1.10 (0.79, 1.52)	0.82 (0.58, 1.15)	0.26	0.93 (0.83, 1.05)		
Model 2	1.00	0.86 (0.61, 1.21)	1.10 (0.80, 1.52)	0.82 (0.58, 1.17)	0.29	0.93 (0.82, 1–05)		
Model 3	1.00	0.83 (0.59, 1.18)	1.03 (0.74, 1.44)	0.77 (0.54, 1.10)	0.15	0.90 (0.80, 1.08)		
Model 4	1.00	0.83 (0.59, 1.18)	1.02 (0.73, 1.43)	0.78 (0.55, 1.11)	0.16	0.90 (0.80, 1.03)		
Pooled*								
Model 1	1.00	0.69 (0.53, 0.90)	0.86 (0.67, 1.11)	0.66 (0.51, 0.87)	< 0.001	0.85 (0.78, 0.94)		
Model 2	1.00	0.69 (0.53, 0.90)	0.87 (0.67, 1.12)	0.66 (0.50, 0.87)	< 0.001	0.86 (0.78, 0.94)		
Model 3	1.00	0.66 (0.51, 0.87)	0.81 (0.62, 1.04)	0.64 (0.48, 0.84)	0.002	0.82 (0.74, 0.92)		
Model 4	1.00	0.67 (0.51, 0.88)	0.80 (0.62, 1.05)	0.65 (0.49, 0.86)	0.006	0.84 (0.76, 0.92)		

Model 1: logistic regression model adjusted for age and sex

Model 2: as in Model 1 and additionally adjusted for educational level (primary, secondary and university), smoking status (current, former and never smoker), leisure-time physical activity (METs-h/week), and time spent watching TV (h/week)

Model 3: as in Model 2 and additionally adjusted for energy intake (kcal/day), and total protein intake (kg/day)

Model 4: as in Model 3 and additionally adjusted for morbidity (musculoskeletal disease, cardiovascular diseases, cancer, chronic lung disease, and depression)

*Pooled data: models combined using a random-effects model

## Discussion

In this study, we found that a higher adherence to two healthy diet patterns decreases the odds of malnutrition among community-dwelling older adults. These results are independent of the amount of energy and protein intake and held after adjustment for chronic diseases. Our results showed that a hypothetical increase of about 2 points in adherence to the Mediterranean diet and 10 points to the AHEI-2010 is linked to a 16% lower likelihood of malnutrition. A high consumption of fish, vegetables, long chain n-3 fatty acids, and a low consumption of trans fats and sweetened beverages might drive these associations; however, the overall quality of the diet was strongly associated with malnutrition, in comparison with their individual components. The pooling of results from 2 different studies, with heterogeneous populations but with similar data collection methods, allowed for a greater external validity of the associations found.

Around 10% of older adults living in the community suffered from malnutrition (9.47% in the SE-1; 11.65% in the SE-2), which is consistent with other studies that used the GLIM criteria to diagnose malnutrition in study populations like ours [6]. By contrast, in a representative sample of community-dwelling older adults in Israel, malnutrition prevalence was lower (3.4%); this can be explained because they used a more stringent BMI cut off than our study (< 20 kg/m^2^ for all age groups vs. <20 for those under 70 and < 22 for those with 70 or more years) [8]. In studies where the participants were hospitalized or institutionalized, the prevalence was higher (between 20 and 30%) [41, 42], results like those obtained with simple screening tools, such as the “MUST”, to detect multimorbidity in community-dwelling adults [43].

Only a single study has previously assessed diet quality in relation to malnutrition, as defined with the GLIM criteria. This is a recently published cross-sectional analysis among Chinese community-dwelling older adults [23], where malnutrition was inversely related to the Dietary Quality Index International (DQI-I) score, a diet pattern characterized by a high consumption of vegetables and fruits and low consumption of meat and fish. By contrast with our results, this study did not find an association between the Mediterranean Diet Score (MDS) and malnutrition; this may be due to the differences in the Mediterranean diet pattern used [44], as well as a different adjustment for sociodemographic and lifestyle confounders, as well as other intrinsic characteristics of this Asian population.

Several studies have examined diet quality in relation to definitions of malnutrition other than GLIM. In a cross-sectional analysis of data from the Hellenic Longitudinal Investigation of Aging and Diet study, with urban-dwelling participants, lower adherence to the Mediterranean diet was associated with higher nutritional risk, defined with the *Determine Your Nutritional Health* checklist, which includes easy-to-obtain warning signs for poor nutrition: having a disease that affect diet, eating poorly, tooth loss/mouth pain, economic hardship, reduced social contact, taking multiple medicines, involuntary weight loss/gain, need assistance in self-care, and age > 80 [24]; however, no phenotypic criteria were included in this definition. In another small cross-sectional study, authors found that a low-nutrient-dense cluster of foods identified in rural older adults was associated with higher odds of obesity and low nutrient intakes [25]. Data from the Health, Aging, and Body Composition Study, with community-dwelling older adults, examined the association between the Healthy Eating Index and incidence of protein-energy malnutrition, based on low BMI and involuntary weight loss. No association was found after a follow-up of 3 to 4 years [26]. Lastly, in the Healthy Aging in Neighborhoods of Diversity across the Life Span, longitudinal data suggested that diet quality, as measured with a “mean adequacy ratio”, did predict the risk for malnutrition, determined by the Mini Nutritional Assessment [27].

There are well-known risk factors for malnutrition, including decreased appetite associated with aging, gastrointestinal malabsorption, oral health problems, decreased olfactory sensitivity [45], and psychosocial factors, such as cognitive impairment, depression, loneliness, and low educational and economic status (risk factors of malnutrition associated with diet patterns) [46]. We adjusted our analyses for many plausible confounders although we lacked information on several of them.

Strengths of this study include the assessment of habitual diet with a validated diet history and the use of two different predefined high-quality diets, in a large sample of community-living older adults. We used the definition of malnutrition using the GLIM criteria, instead of screening tools used in previous association studies. Although this definition has been implemented for use in clinical settings, its use in cohort studies with phenotypic metrics is optimal to identify malnutrition, instead of relying on self-reported questionnaire [47]. In terms of limitations, this is a cross-sectional analysis, therefore, we cannot rule out reverse causality: people with a good nutritional status may also have better general health to be able to prepare high-quality meals. Diet was self-reported; thus, some misreporting and misclassification may exist. We used a definition of the Mediterranean diet adapted to the characteristics of the Spanish diet; however, other definitions have been published. In addition, despite scientific evidence proving the AHEI-2010 is consistently associated with the lower risk of chronic diseases, the score assignment is subjective. Although we adjusted our analyses for many potential confounders, we cannot rule out the influence of those not measured, which may partially explain the differences in the estimates of the association between both cohorts. Lastly, selection bias could also play a role in our results, since participants included and excluded were different in age, sex, and diagnosed comorbidies.

## Conclusion

In conclusion, adhering to the MEDAS and AHEI-2010 diet patterns was associated with less likelihood of malnutrition defined with phenotypic GLIM criteria. Measures to guarantee high diet quality among older adults seem necessary as part of the routine clinical strategy to prevent malnutrition and its consequences.

### Electronic supplementary material

Below is the link to the electronic supplementary material.

Supplementary Material 1

## Data Availability

Data describe in the manuscript, code book, and analytic code will be made available upon request pending application and approval. The datasets used and/or analysed during the current study are available from the corresponding author on reasonable request.

## Abbreviations

AHEI: 2010 Alternative Healthy Eating Index
ANOVA: Analysis of variance
BIA: Bioelectrical impedance analysis
BMI: Body mass index
CI: Confidence Interval
DQI-I: Dietary quality index international score
GLIM: Global Leadership Initiative on Malnutrition
MDS: Mediterranean diet score
MEDAS: Mediterranean Diet Adherence Screener
METs: Metabolic Equivalent Tasks
OR: Odds ratios
SE-1: Seniors-ENRICA-1
SE-2: Seniors-ENRICA-2
SMI: Skeletal muscle mass index
SMM: Skeletal muscle mass
