# Supplementary material for: Association between diet quality and malnutrition: pooled results from two population-based studies in older adults
Source: BMC Geriatr. 2024 May 10;24:417. doi: 10.1186/s12877-024-04984-5 (PMC11088013; doi:10.1186/s12877-024-04984-5)
Supplement: Supplementary file 1 — Supplementary Material 1 [file 12877_2024_4984_MOESM1_ESM.docx]

**TABLE OF CONTENTS SUPPLEMENTARY APPENDIX**

Components and scoring of the dietary quality scores

Table S1. Components and scoring of the MEDAS, and AHEI-2010 diet quality scores.

Table S2. Odds ratios (95% Confidence Interval) of malnutrition according to components of the MEDAS score, pooled data.

Table S3. Odds ratios (95% Confidence Interval) of malnutrition according to components of the AHEI-2010 score, pooled data.

Table S4. Odds ratios (95% Confidence Interval) of malnutrition according to the MEDAS diet quality scores excluding the alcohol component in the Seniors-ENRICA-1, Seniors-ENRICA-2 and pooled data.

Table S5. Odds ratios (95% Confidence Interval) of malnutrition according to quartiles of the AHEI-2010 diet quality scores excluding the alcohol component in the Seniors-ENRICA-1, Seniors-ENRICA-2 and pooled data.

| **Table S1.** Components and scoring of the MEDAS and AHEI-2010 diet quality scores. |
| --- |

| **Component** | | **Minimum score** | **Maximum score** |
| --- | --- | --- | --- |
| MEDAS | | 0 | 1 |
| 1. Using olive oil as main source of fat for cooking | | No | Yes |
| 1. Olive oil (g/day) | | <36 | ≥36 |
| 1. Vegetables (servings/day for low/int./high energy intake) | | <2/2.33/2.67 | ≥2/2.33/2.67 |
| 1. Fruit, including fresh-squeezed juice (servings/day for low/int./high energy intake) | | <3/3.6/4.2 | ≥3/3.6/4.2 |
| 1. Red and processed meats (servings/day for low/int./high energy intake) | | ≥0.8/1/1.2 | <0.8/1/1.2 |
| 1. Butter, margarine or cream (servings/day for low/int./high energy intake) | | ≥0.83/1/1.25 | <0.83/1/1.25 |
| 1. Carbonated or sugar-sweetened beverages (servings/day) | | ≥1 | <1 |
| 1. Wine (servings/week for low/int./high energy intake) | | <4.67/5.83/7 | ≥4.67/5.83/7 |
| 1. Legumes (servings/week for low/int./high energy intake) | | <3/3.5/4 | ≥3/3.5/4 |
| 1. Fish or seafood (servings/week for low/int./high energy intake) | | <3/3.76/4.5 | ≥3/3.76/4.5 |
| 1. Commercial pastries (servings/week for low/int./high energy intake) | | ≥2/3/4 | <2/3/4 |
| 1. Nuts (servings/week for low/int./high energy intake) | | <2/2.5/3 | ≥2/2.5/3 |
| 1. Preferring white meat over red meat | | No | Yes |
| 1. Dishes with a traditional sauce of tomatoes, garlic, onion or leeks sautéed in olive oil (servings/week) | | <2 | ≥2 |
| Total | | 0 | 14 |
| AHEI-2010 | | 0 | 10 |
| 1. Vegetables, excluding potatoes (servings/day) | | 0 | ≥5 |
| 1. Fruit (servings/day) | | 0 | ≥4 |
| 1. Whole grains (g/day) | Men | 0 | ≥90 |
|  | Women | 0 | ≥75 |
| 1. Nuts and legumes (servings/day) | | 0 | ≥1 |
| 1. Long-chain n-3 fats (EPA+DHA) (mg/day) | | 0 | ≥250 |
| 1. Polyunsaturated fatty acids (% of energy intake) | | ≤2 | ≥10 |
| 1. Sugar-sweetened beverages and fruit juice (servings/day) | | ≥1 | 0 |
| 1. Red and processed meats (servings/day) | | ≥1.5 | 0 |
| 1. Trans fat (% of energy intake) | | ≥0 | ≤0.5 |
| 1. Sodium (mg/day) | | Highest decile | Lowest decile |
| 1. Alcohol (g/day) | Men | ≥35 | 5-10 |
|  | Women | ≥25 | 5-15 |
| Total | | 0 | 110 |

For the scoring of MEDAS: 1 serving of vegetables = 150 g; 1 serving of fruit = 125 g; 1 serving of red/processed meats = 125 g; 1 serving of butter, margarine or cream = 12 g; 1 serving of carbonated or sugar-sweetened beverages = 200 mL; 1 serving of wine = 100 mL; 1 serving of legumes = 150 g; 1 serving of fish or seafood = 100 g; 1 serving of commercial pastries = 50 g; 1 serving of nuts = 30 g.

For the scoring of AHEI-2010: Intakes between the minimum (0) and maximum (10) levels are scored proportionately. 1 serving of vegetables = 0.5 cups of vegetables or 1 cup of green leafy vegetables; 1 serving of fruit = 160 g; 1 serving of sugar-sweetened beverages and fruit juice = 8 oz; 1 serving of red/processed meats = 4 oz of unprocessed meat or 1.5 oz of processed meat (1 cup = 236.59 g; 1 oz = 28.35 g). Values in lowest decile of sodium were ≤1704 mg/day in men and ≤1276 mg/day in women, and in highest decile were ≥4735 mg/day in men and ≥3833 mg/day in women. Non-drinkers received a score of 2.5.

AHEI = Alternate Healthy Eating Index; MEDAS = Mediterranean Diet Adherence Screener; MDS = Mediterranean Diet Score.

| **Table S2.** Odds ratios (95% confidence interval) of malnutrition according to components of the MEDAS score, pooled data. | | |
| --- | --- | --- |
| MEDAS components | 0 | 1 |
| Using olive oil as main source of fat for cooking | 1.00 | 0.92 (0.69-1.22) |
| Olive oil | 1.00 | 1.16 (0.83-1.61) |
| Vegetables | 1.00 | 0.78 (0.58-1.03) |
| Fruit, including fresh-squeezed juice | 1.00 | 0.81 (0.65-1.00) |
| Red and processed meats | 1.00 | 1.28 (0.92-1.79) |
| Butter, margarine or cream | 1.00 | 0.71 (0.48-1.06) |
| Carbonated or sugar-sweetened beverages | 1.00 | 0.56 (0.41-0.78) |
| Wine | 1.00 | 1.05 (1.02-1.07) |
| Legumes | 1.00 | 1.23 (0.92-1.66) |
| Fish or seafood | 1.00 | 0.62 (0.50-0.77) |
| Commercial pastries | 1.00 | 0.85 (0.70-1.03) |
| Nuts | 1.00 | 1.05 (1.03-1.07) |
| Preferring white meat over red meat | 1.00 | 1.00 (0.80-1.24) |
| Dishes with a traditional sauce of tomatoes, garlic, onion or leeks sautéed in olive oil | 1.00 | 0.86 (0.66-1.12) |

Logistic regression model adjusted for age, sex, educational level (primary, secondary and university), smoking status (current, former and never former), leisure-time physical activity (METs-h/week), time spent watching TV (h/week), energy intake (kcal/day), total protein intake (kg/day), and morbidity (musculoskeletal disease, cardiovascular diseases, cancer, chronic lung disease and depression), as well as for each of the other food components of the index. Data from both studies were pooled using a random-effects model.

| **Table S3.** Odds ratios (95% confidence interval) of malnutrition according to components of the AHEI-2010 score, pooled data. | |
| --- | --- |
| AHEI-2010 components | Per 2 points increment^1^ |
| Vegetables, excluding potatoes | 0.83 (0.73-0.93) |
| Fruit | 0.90 (0.76-1.06) |
| Whole grains | 1.06 (0.88-1.29) |
| Nuts and legumes | 0.89 (0.82-0.98) |
| Long chain n-3 fats | 0.83 (0.76-0.90) |
| Polyunsaturated fatty acids | 0.99 (0.91-1.07) |
| Sugar-sweetened beverages and fruit juice | 0.92 (0.86-0.97) |
| Red and processed meats | 1.08 (1.01-1.15) |
| Trans fats | 0.79 (0.67-0.93) |
| Sodium | 0.99 (0.88-1.10) |
| Alcohol | 0.94 (0.88-1.00) |

Logistic regression model adjusted for age, sex, educational level (primary, secondary and university), smoking status (current, former and never former), leisure-time physical activity (METs-h/week), time spent watching TV (h/week), energy intake (kcal/day), total protein intake (kg/day), and morbidity (musculoskeletal disease, cardiovascular diseases, cancer, chronic lung disease and depression), ), as well as for each of the other food components of the index. Data from both studies were pooled using a random-effects model.

^1^An increase in points represents a lower intake for the unhealthy components: sodium, sugar-sweetened beverages and red and processed meat.

| **Table S4**. Odds ratios (95% confidence interval) of malnutrition according to adherence to the MEDAS diet quality score excluding the alcohol component in the Seniors-ENRICA-1, Seniors-ENRICA-2 and pooled data. | | | | | | | | |  |
| --- | --- | --- | --- | --- | --- | --- | --- | --- | --- |
|  | Adherence to the MEDAS score | | |  | |  |  | |  |
|  | Low | Moderate | High | | | *p* trend | Per 1-SD increment in MEDAS | |  |
|  | <7 points | 7-9 points | ≥9 points | | |  |  | |  |
| Seniors-ENRICA-1 (n=1921) | | | | |  | | |  |  |
| N | 623 | 882 | 416 | | |  |  | |  |
| n cases | 72 | 86 | 24 | | |  |  | |  |
| Model 1 | 1.00 | 0.84 (0.60-1.17) | 0.50 (0.31-0.80) | | | 0.007 | 0.81 (0.69-0.94) | |  |
| Model 2 | 1.00 | 0.85 (0.61-1.19) | 0.51 (0.32-0.84) | | | 0.01 | 0.82 (0.70-0.96) | |  |
| Model 3 | 1.00 | 0.81 (0.57-1.14) | 0.45 (0.27-0.74) | | | 0.003 | 0.78 (0.66-0.92) | |  |
| Model 4 | 1.00 | 0.81 (0.58-1.15) | 0.44 (0.26-0.72) | | | 0.002 | 0.77 (0.66-0.91) | |  |
| Seniors-ENRICA-2 (n=2652) | | | | | | | | |  |
| N | 919 | 1166 | 567 | | |  |  | |  |
| n cases | 113 | 140 | 56 | | |  |  | |  |
| Model 1 | 1.00 | 0.96 (0.74-1.24) | 0.81 (0.57-1.17) | | | 0.36 | 0.95 (0.84-1.07) | |  |
| Model 2 | 1.00 | 0.96 (0.74-1.24) | 0.80 (0.56-1.16) | | | 0.36 | 0.94 (0.83-1.06) | |  |
| Model 3 | 1.00 | 0.89 (0.68-1.15) | 0.75 (0.51-1.08) | | | 0.17 | 0.91 (0.81-1.03) | |  |
| Model 4 | 1.00 | 0.87 (0.67-1.13) | 0.74 (0.51-1.07) | | | 0.14 | 0.91 (0.80-1.02) | |  |
| Pooled* |  |  |  | | |  |  | |  |
| Model 1 | 1.00 | 0.91 (0.75-1.12) | 0.68 (0.51-0.90) | | | 0.008 | 0.89 (0.81-0.98) | |  |
| Model 2 | 1.00 | 0.92 (0.75-1.13) | 0.68 (0.51-0.91) | | | 0.009 | 0.89 (0.81-0.98) | |  |
| Model 3 | 1.00 | 0.86 (0.70-1.06) | 0.63 (0.46-0.85) | | | 0.002 | 0.86 (0.78-0.95) | |  |
| Model 4 | 1.00 | 0.85 (0.69-1.04) | 0.62 (0.46-0.83) | | | 0.002 | 0.86 (0.78-0.94) | |  |
| \| Model 1: logistic model adjusted for age and sex. \|  \|  \|  \|  \| \| --- \| --- \| --- \| --- \| --- \| \| Model 2: logistic model adjusted as in Model 1 and for educational level (primary, secondary and university), smoking status (current, former and never smoker), leisure-time physical activity (METs-h/week), and time spent watching TV (h/week).  Model 3: logistic model adjusted as in Model 2 and for energy intake (kcal/day) and total protein intake (kg/day). \| \| \| \| \| \| Model 4: logistic model adjusted as in Model 3 and for morbidity (musculoskeletal disease, cardiovascular diseases, cancer, chronic lung disease, and depression). \| \| \| \| \| \| *Pooled data: results from the multivariable-adjusted models combined using a random-effects model. \| \| \| \| \|   1-SD increment in the MEDAS: Seniors-ENRICA-1: 1.6; Seniors-ENRICA-2: 1.7. | | | | | | | | |  |

**Table S5**. Odds Ratio (95% Confidence Interval) of malnutrition according to quartiles of adherence to the AHEI-2010 diet quality score without the alcohol component in the Seniors-ENRICA-1, Seniors-ENRICA-2 and pooled data.

|  | Adherence to the AHEI-2010 | | | | | | |  | |  | |  |
| --- | --- | --- | --- | --- | --- | --- | --- | --- | --- | --- | --- | --- |
|  | Quartile 1 | Quartile 2 | Quartile 3 | | | Quartile 4 | | *p* trend | | Per 1-SD increment in AHEI-2010 | |  |
| Seniors-ENRICA-1 (n=1921) | |  |  | | |  | |  | |  | |  |
| Range | <56.1 | 56.1-62.9 | 63.0-69.3 | | | ≥69.4 | |  | |  | |  |
| N | 481 | 480 | 480 | | | 480 | |  | |  | |  |
| n cases | 63 | 42 | 39 | | | 38 | |  | |  | |  |
| Model 1 | 1.00 | 0.61 (0.40-0.92) | 0.55 (0.36-0.84) | | | 0.55 (0.36-0.84) | | <0.001 | | 0.75 (0.65-0.88) | |  |
| Model 2 | 1.00 | 0.61 (0.40-0.93) | 0.54 (0.35-0.83) | | | 0.56 (0.37-0.87) | | <0.001 | | 0.75 (0.64-0.88) | |  |
| Model 3 | 1.00 | 0.61 (0.40-0.93) | 0.53 (0.34-0.81) | | | 0.56 (0.36-0.86) | | <0.001 | | 0.74 (0.64-0.87) | |  |
| Model 4 | 1.00 | 0.62 (0.41-0.95) | 0.54 (0.35-0.84) | | | 0.56 (0.36-0.86) | | <0.001 | | 0.75 (0.64-0.87) | |  |
| Seniors-ENRICA-2 (n=2652) | |  |  | | |  | |  | |  | |  |
| Range | <56.2 | 56.2-62.9 | 63.0-69.3 | | | ≥69.4 | |  | |  | |  |
| N | 663 | 663 | 663 | | | 663 | |  | |  | |  |
| n cases | 81 | 72 | 88 | | | 68 | |  | |  | |  |
| Model 1 | 1.00 | 0.86 (0.61-1.20) | 0.90 (0.64-1.26) | | | 1.02 (0.74-1.42) | | 0.75 | | 0.98 (0.87-1.11) | |  |
| Model 2 | 1.00 | 0.86 (0.61-1.20) | 0.90 (0.61-1.20) | | | 1.04 (0.74-1.45) | | 0.82 | | 0.98 (0.87-1.10) | |  |
| Model 3 | 1.00 | 0.85 (0.60-1.21) | 0.83 (0.59-1.18) | | | 0.99 (0.70-1.39) | | 0.52 | | 0.95 (0.84-1.07) | |  |
| Model 4 | 1.00 | 0.86 (0.61-1.22) | 0.83 (0.58-1.17) | | | 0.99 (0.71-1.40) | | 0.52 | | 0.95 (0.84-1.07) | |  |
| Pooled* |  |  |  | | |  | |  | |  | |  |
| Model 1 | 1.00 | 0.75 (0.58-0.98) | 0.74 (0.57-0.97) | | | 0.81 (0.63-1.05) | | 0.003 | | 0.88 (0.80-0.97) | |  |
| Model 2 | 1.00 | 0.75 (0.58-0.98) | 0.74 (0.57-0.97) | | | 0.82 (0.63-1.07) | | 0.003 | | 0.98 (0.96-1.01) | |  |
| Model 3 | 1.00 | 0.74 (0.57-0.97) | 0.70 (0.53-0.91) | | | 0.80 (0.61-1.04) | | <0.001 | | 0.86 (0.79-0.95) | |  |
| Model 4 | 1.00 | 0.75 (0.58-0.98) | 0.70 (0.53-0.92) | | | 0.80 (0.61-1.04) | | 0.002 | | 0.87 (0.79-0.95) | |  |
| Model 1: logistic model adjusted for age and sex. | | | |  |  | |  | |  | |  | |
| Model 2: logistic model adjusted as in Model 1 and for educational level (primary, secondary and university), smoking status (current, former and never smoker), leisure-time physical activity (METs-h/week), and time spent watching TV (h/week).  Model 3: logistic model adjusted as in Model 2 and for energy intake (kcal/day), and total protein (kg/day). | | | | | | | | | | | | |
| Model 4: logistic model adjusted as in Model 3 and morbidity (musculoskeletal disease, cardiovascular diseases, cancer, chronic lung disease, and depression). | | | | | | | | | | | | |
| *Pooled data: Results from the multivariable-adjusted models combined using a random-effects model. | | | | | | | | |  | |  | |

1-SD increase in AHEI-2010: Seniors-ENRICA-1: 9.9; Seniors-ENRICA-2-2: 9.4.
